# Supplementary material for: Mild and moderate COVID-19 during Alpha, Delta and Omicron pandemic waves in urban Maputo, Mozambique, December 2020-March 2022: A population-based surveillance study
Source: PLOS Glob Public Health. 2024 Aug 5;4(8):e0003550. doi: 10.1371/journal.pgph.0003550 (PMC11299809; doi:10.1371/journal.pgph.0003550)
Supplement: S1 Table — (DOCX) [file pgph.0003550.s002.docx]

# Supporting information of Mild and moderate COVID-19 during Alpha, Delta and Omikron pandemic waves in urban Maputo, Mozambique, December 2020-March 2022: a population-based surveillance study

*S1 Table. Infection- and vaccine-induced SARS-CoV-2 seroprevalence based on detection of antibodies against different SARS-CoV-2 antigens*

| **month** | **antibody detection** | **RBP and NP positive** | | | | **two out of RBD, NP and S1S2 positive** | | | | **RBD positive (regardless of S1S2 and NP)** | | | |
| --- | --- | --- | --- | --- | --- | --- | --- | --- | --- | --- | --- | --- | --- |
|  |  | **n** | **prop** | **95%CI** | | **n** | **prop** | **95%CI** | | **n** | **prop** | **95%CI** | |
| 2020-12 | negative | 118 | 0.952 | 0.914 | 0.989 | 112 | 0.903 | 0.851 | 0.955 | 111 | 0.895 | 0.841 | 0.949 |
| 2020-12 | positive after infection | 6 | 0.048 | 0.011 | 0.086 | 12 | 0.097 | 0.045 | 0.149 | 13 | 0.105 | 0.051 | 0.159 |
| 2021-01 | negative | 433 | 0.945 | 0.925 | 0.966 | 416 | 0.908 | 0.882 | 0.935 | 399 | 0.871 | 0.840 | 0.902 |
| 2021-01 | positive after infection | 25 | 0.055 | 0.034 | 0.075 | 42 | 0.092 | 0.065 | 0.118 | 59 | 0.129 | 0.098 | 0.160 |
| 2021-02 | negative | 205 | 0.911 | 0.874 | 0.948 | 195 | 0.867 | 0.822 | 0.911 | 192 | 0.853 | 0.807 | 0.900 |
| 2021-02 | positive after infection | 20 | 0.089 | 0.052 | 0.126 | 30 | 0.133 | 0.089 | 0.178 | 33 | 0.147 | 0.100 | 0.193 |
| 2021-03 | negative | 465 | 0.884 | 0.857 | 0.911 | 447 | 0.850 | 0.819 | 0.880 | 436 | 0.829 | 0.797 | 0.861 |
| 2021-03 | positive after infection | 59 | 0.112 | 0.085 | 0.139 | 77 | 0.146 | 0.116 | 0.177 | 88 | 0.167 | 0.135 | 0.199 |
| 2021-03 | positive after vaccination | 2 | 0.004 | -0.001 | 0.009 | 2 | 0.004 | -0.001 | 0.009 | 2 | 0.004 | -0.001 | 0.009 |
| 2021-04 | negative | 244 | 0.758 | 0.711 | 0.805 | 238 | 0.739 | 0.691 | 0.787 | 212 | 0.658 | 0.607 | 0.710 |
| 2021-04 | positive after infection | 75 | 0.233 | 0.187 | 0.279 | 81 | 0.252 | 0.204 | 0.299 | 107 | 0.332 | 0.281 | 0.384 |
| 2021-04 | positive after vaccination | 3 | 0.009 | -0.001 | 0.020 | 3 | 0.009 | -0.001 | 0.020 | 3 | 0.009 | -0.001 | 0.020 |
| 2021-05 | negative | 181 | 0.733 | 0.678 | 0.788 | 179 | 0.725 | 0.669 | 0.780 | 163 | 0.660 | 0.601 | 0.719 |
| 2021-05 | positive after infection | 62 | 0.251 | 0.197 | 0.305 | 64 | 0.259 | 0.204 | 0.314 | 79 | 0.320 | 0.262 | 0.378 |
| 2021-05 | positive after vaccination | 4 | 0.016 | 0.000 | 0.032 | 4 | 0.016 | 0.000 | 0.032 | 5 | 0.020 | 0.003 | 0.038 |
| 2021-06 | negative | 48 | 0.658 | 0.549 | 0.766 | 46 | 0.630 | 0.519 | 0.741 | 42 | 0.575 | 0.462 | 0.689 |
| 2021-06 | positive after infection | 23 | 0.315 | 0.209 | 0.422 | 25 | 0.342 | 0.234 | 0.451 | 29 | 0.397 | 0.285 | 0.510 |
| 2021-06 | positive after vaccination | 2 | 0.027 | -0.010 | 0.065 | 2 | 0.027 | -0.010 | 0.065 | 2 | 0.027 | -0.010 | 0.065 |
| 2021-07 | negative | 143 | 0.665 | 0.602 | 0.728 | 143 | 0.665 | 0.602 | 0.728 | 115 | 0.535 | 0.468 | 0.602 |
| 2021-07 | positive after infection | 61 | 0.284 | 0.223 | 0.344 | 61 | 0.284 | 0.223 | 0.344 | 86 | 0.400 | 0.335 | 0.465 |
| 2021-07 | positive after vaccination | 11 | 0.051 | 0.022 | 0.081 | 11 | 0.051 | 0.022 | 0.081 | 14 | 0.065 | 0.032 | 0.098 |

RBD= receptor-binding domain (of the spike protein), NP=nucleocapsid protein, S1S2=spike glycoprotein S1 and S2 subunit
