# Supplementary material for: Mild and moderate COVID-19 during Alpha, Delta and Omicron pandemic waves in urban Maputo, Mozambique, December 2020-March 2022: A population-based surveillance study
Source: PLOS Glob Public Health. 2024 Aug 5;4(8):e0003550. doi: 10.1371/journal.pgph.0003550 (PMC11299809; doi:10.1371/journal.pgph.0003550)
Supplement: Attachment — Submitted filename: PGPH_C19.docx [file pgph.0003550.s003.docx]

Review of

Mild and moderate COVID-19 during Alpha, Delta and Omikron pandemic waves in urban Maputo, Mozambique, December 2020-March 2022: a population-based surveillance study

General comments:

This paper presents a epidemiological study about mild and moderate COVID-19 occurrence in Mozambique proceeded through a population-based surveillance

General comments

- Thank you for stating the following financial disclosure and Acknowledgments Section and to provide a repository information for your data, codes and maps.
- Please, check if the keywords were included in DeCS/MeSH

Introduction

- Thanks for the introduction, it is very well written, easy to read and presents what is necessary on the topic. Authors may choose to discreetly include information about study locations, for example, population size.
- Please check grammar of sentence in third paragraph. Use commas to numbers.
- I understood that in Sep 2021 5% received at least 1 dose, and that 6 months later there was an increase to 40% with 1 dose, is this correct? It would be good to place more emphasis on vaccination coverage.
- Why Omicron was spelled with K, not C (for English)?

Methods

- In the second paragraph it would be delimited as a section "data collection" better describing the tools for the two studies (i) socio-economic questionnaire, (ii) questionnaire to collect symptoms and (iii) self-administered biological samples. Please also explain whether the questionnaires were different for each age group.
- Were questions about vaccination collected or did population coverage be used to interpret the results?
- I suggest that the authors describe better the definition of suspected cases (possible cases) including a sentence answering this question: What happens if the covid case is not confirmed, is it a negative PCR that was used to confirm the case or rule it out? Even with a well-reported clinic? see an example of how well you defined seropositivity (line 110). Even though the data analysis section perfectly indicates procedures to do that, it would be good to define it in a simpler way here.
- It was not clear to me whether, when there was a positive case, other residents of the same residence were tested even if they were asymptomatic, close contact was not a possible case? Can you explain this better here, in the methods section before appears in the next sections?
- Can you explain why close contact was not a possible case? I understand that this is mild and moderate covid, but it would be nice to inform you if there were asymptomatic cases or if this was not considered in this study. As you pointed in the discussion.

Laboratory procedures

- Laboratory procedures is a little difficult to follow because it is a very long paragraph, I suggest dividing and removing unnecessary punctuation that divides sentences like ( : ) on line 100.
- I missed the reference of the collections in each study, is difficult to follow, especially in time.
- What was done with the samples after they were tested? Stored? for how long, destroyed?
- Line 99, move citation to reference, following the journal's rules. Name (year) [ref]

Data analysis

- It's very good! It's very good! I only have a question, is the prevalence adjusted for the tests sensitivity/specificity?
- Previous, the methods need specifying better the outcomes. Define and re-specify your outcomes, presenting all your variables in clear terms.

Results

- You can include a comma in the numbers presented in the text, it is easier to read. (line 149-153)
- HIV diagnosis or comorbidities were self-reported? Please include this information in the methods section as a case definition.
- In line 151-152, I don't understand who recorded socio-economic status, does information only apply to over 18yo for example? Please move this information, clarifying this doubt, to the methods section. Please answer the following question very simply: Considering that the study includes people of all ages, are the educational level results for those over 18? (line152-153)
- Figure 1, can you explain why some people were not tested?
- Please, include in all foot tables the name of the test used in the presented aOR, OR, HR (...)
- Table 1 - replace throat pain for sore throat.
- Figure 2, It is not very clear if “positive after vaccination” refers to vaccine-induced antibody +, please, consider replace the text for meaning is clear.
- Line 181 there is no need to parentheses.

Discussion/Conclusion

- Thanks for the discussion section, its drawn appropriately based on the data presented.
- Please remove the parentheses of (symptomatic) on line 219, the sentence is confusing.
- Review line 253
- Did the authors observe any limitation? It would be nice to comment on in the discussion section. Please explain whether these limitations have been mitigated and how you plan to address them. Otherwise, authors can discuss any participation issue in the limitations section.
- There are few spelling errors that need to be revised.
