# Supplementary material for: Mild and moderate COVID-19 during Alpha, Delta and Omicron pandemic waves in urban Maputo, Mozambique, December 2020-March 2022: A population-based surveillance study
Source: PLOS Glob Public Health. 2024 Aug 5;4(8):e0003550. doi: 10.1371/journal.pgph.0003550 (PMC11299809; doi:10.1371/journal.pgph.0003550)
Supplement: Attachment — Submitted filename: Response to Reviewers_mvds.docx [file pgph.0003550.s004.docx]

Rebuttal letter accompanying the review of PGPH-D-24-00678
Mild and moderate COVID-19 during Alpha, Delta and Omikron pandemic waves in urban Maputo, Mozambique, December 2020-March 2022: a population-based surveillance study

Dear Editor, dear reviewers,

Thank you for the review and the encouraging comments. We addressed comments and provide below a point-by-point rebuttal, in bold.

Kind regards,

Brecht Ingelbeen

Institute of Tropical Medicine, Antwerp, Belgium

*Journal Requirements*

1. Please provide separate figure files in .tif or .eps format.

**Provided now. Please note that I uploaded all four plots belonging to figure 1 individually.**

2. Please include a complete copy of PLOS’ questionnaire on inclusivity in global research in your revised manuscript.

**We completed the questionnaire and submitted it now.**

3. Please review your reference list to ensure that it is complete and correct. If you have cited papers that have been retracted, please include the rationale for doing so in the manuscript text, or remove these references and replace them with relevant current references. Any changes to the reference list should be mentioned in the rebuttal letter that accompanies your revised manuscript. If you need to cite a retracted article, indicate the article’s retracted status in the References list and also include a citation and full reference for the retraction notice.

**Checked, and no changes needed**

*Additional Editor Comments*

The study provides valuable insight into SARS-CoV-2 in Mozambique where there is a relatively paucity of COVID-19 epidemiological data. The reviewers had provides valuable comment which though minor, can significantly strengthen the manuscript.

Please pay particular attention to the following area when addressing the reviewer's comments:

1. Sampling strategy and its impact on interpretation of finding.

**We added a subsection sampling strategy to the methods, with some changes in the data collection subsection to avoid repeating procedures. I added its impact to the new limitations subsection in the discussion.**

2. In discussion, the difference of the wave/epi pattern of Mozambique with its neighbouring countries warrant some comments.

**We elaborated now in more detail in the second paragraph of the discussion. We did however remain careful overinterpreting somewhat conflicting conclusions one could draw from different findings: (1) short peaks in COVID-19 detected through active surveillance indicating widespread transmission, without any observable temporal effect of introduced measures/interventions that could have slowed transmission, versus (2) the lower sero-prevalence compared to some other settings despite a more sensitive serological assay used, coherence with the national COVID-19 surveillance reporting few cases and low bed occupancy in the first year of the pandemic, and coherence in relative differences in sero-prevalence (e.g., over time or between age groups). We have had lengthy discussions with the entire team on the comparison with other countries in the region, and with the evidence we have opted to not draw stronger conclusions. With serological testing is completed, samples of the first six months can still be retested on a newly installed Bioplex 200 platform at INS when resources are available. This should provide quantitative serological results, which could provide further insights in waning immunity and re-infection. Such additional analyses could also support the interpretation of the sero-prevalence we reported here.**

3. A robust study limitations section, particularly related to sampling strategy, is needed.

**Added a subsection limitations (five major limitations) to the discussion, reporting limitations, how these (might) affect results and how this has been addressed.**

*Comments to the Author*
Reviewer #1: 
General comments:
This paper presents a epidemiological study about mild and moderate COVID-19 occurrence in Mozambique proceeded through a population-based surveillance
General comments
⎫ Thank you for stating the following financial disclosure and Acknowledgments Section and to provide a repository information for your data, codes and maps.

**We now added those sections after the acknowledgements, in addition to the information already provided in the Additional Information section on the submission page of editorial manager.**

⎫ Please, check if the keywords were included in DeCS/MeSH
**We did check that keywords matched MeSH terms, and replaced one (others were MeSH): Population-based surveillance by Population Surveillance* / methods.**

Introduction
⎫ Thanks for the introduction, it is very well written, easy to read and presents what is necessary on the topic. Authors may choose to discreetly include information about study locations, for example, population size. **We added the 2020 population size of Mozambique and of Maputo city.**
⎫ Please check grammar of sentence in third paragraph. Use commas to numbers.
⎫ I understood that in Sep 2021 5% received at least 1 dose, and that 6 months later there was an increase to 40% with 1 dose, is this correct? It would be good to place more emphasis on vaccination coverage. **These numbers are to provide some general context to the country situation, and are based on country-wide data (available on ourworldisdata.org). More detailed evolution of vaccination status/coverage within the study participants is provided in the section reporting sero-prevalence and the corresponding figure.**
⎫ Why Omicron was spelled with K, not C (for English)? **Indeed, apologies, this is now corrected**

Methods
⎫ In the second paragraph it would be delimited as a section "data collection" better describing the tools for the two studies (i) socio-economic questionnaire, (ii) questionnaire to collect symptoms and (iii) self-administered biological samples. Please also explain whether the questionnaires were different for each age group. **We added a subsection explaining data collection, more clearly distinguishing between different forms and databases.**
⎫ Were questions about vaccination collected or did population coverage be used to interpret the results? **COVID-19 vaccination status was recorded among sero-survey participants and possible cases. Vaccine-induced sero-prevalence we report on is based on seroconversion following reported vaccination, of which we recorded dates of the administration of vaccines after checking vaccination cards.**
⎫ I suggest that the authors describe better the definition of suspected cases (possible cases) including a sentence answering this question: What happens if the covid case is not confirmed, is it a negative PCR that was used to confirm the case or rule it out? Even with a well-reported clinic? see an example of how well you defined seropositivity (line 110). Even though the data analysis section perfectly indicates procedures to do that, it would be good to define it in a simpler way here. **We added a definition of a possible COVID-19 case, which was indeed not stated explicitly. As we conducted population-based surveillance, sensitivity of self-administered nasal swabs will not be perfect, yet we believe trends and relative differences between confirmed and discarded cases are sufficient for our study objectives (describing transmission and risk, rather than an estimation of cumulative burden of COVID-19 cases or deaths) and to relate to other research. Even if the number of cases is underestimated, it implies an observed association could be smaller than reality. We added some considerations when interpreting the data to the limitations section and clarified to the extent possible the distinction in the method section.**
⎫ It was not clear to me whether, when there was a positive case, other residents of the same residence were tested even if they were asymptomatic, close contact was not a possible case? Can you explain this better here, in the methods section before appears in the next sections? **Testing of household contacts is not included in the analyses presented in this manuscript. Household investigations, testing close contacts of cases for active infection and with serological assays for past infections or immunity, was intended to be part of the research project. However, because COVID-19 peaks were so pronounced, the work and testing load during such peak periods for the regular services were at its maximum, which did not allow to add additional visits and samples for those household investigations.**

⎫ Can you explain why close contact was not a possible case? I understand that this is mild and moderate covid, but it would be nice to inform you if there were asymptomatic cases or if this was not considered in this study. As you pointed in the discussion.
**We agree, but as explained with the previous question: most COVID-19 cases occurred during limited peaks, when testing capacity was at its maximum capacity. Even though it was an initial objective of the research project, we were forced to suspend that component.**

Laboratory procedures

⎫ Laboratory procedures is a little difficult to follow because it is a very long paragraph, I suggest dividing and removing unnecessary punctuation that divides sentences like ( : ) on line 100. ⎫ I missed the reference of the collections in each study, is difficult to follow, especially in time. **We added clarification at the start of each paragraph what component of the surveillance the procedures are for.**

⎫ What was done with the samples after they were tested? Stored? for how long, destroyed? **We added a phrase: “Dried blood spot cards containing unused blood spots and aliquots of nasal swab samples are stored in -80°C freezers at Instituto Nacional de Saúde and will be destroyed after five years.”**

⎫ Line 99, move citation to reference, following the journal's rules. Name (year) [ref] **Thanks for noticing. We corrected this.**

Data analysis
⎫ It's very good! It's very good! I only have a question, is the prevalence adjusted for the tests sensitivity/specificity? **No the prevalence is not corrected because there is no valid or robust correction factor we could use for that. Sensitivity reported in the validation test done by our colleagues (Mariën et al, Journal of Virological Methods, 2021) used samples collected under different circumstances. Moreover, sensitivity differed between mild versus severe/hospitalised cases. Deducting correction factors by severity of prior reported illness episodes (or no illness if asymptomatic infection) would not be based on sound evidence, and would have overcomplicated interpretation of results. We instead opted to report the prevalence based on different antigen targets, which allowed comparing with (non corrected) sero-surveys reported elsewhere, mostly using commercial assays, including those in the systematic review by the WHO.**
⎫ Previous, the methods need specifying better the outcomes. Define and re-specify your outcomes, presenting all your variables in clear terms. **Thanks. We checked and made sure all exposure and outcome variables used for the analyses are now clearly defined in the data collection subsection, and added those still missing.**

Results
⎫ You can include a comma in the numbers presented in the text, it is easier to read. (line 149-153) **Thank you, we have adapted as suggested.**

⎫ HIV diagnosis or comorbidities were self-reported? Please include this information in the methods section as a case definition. **Correct, these were self-reported, we have now added this.**
⎫ In line 151-152, I don't understand who recorded socio-economic status, does information only apply to over 18yo for example? Please move this information, clarifying this doubt, to the methods section. **Socio-economic status was recorded within the HDSS and applies to the households included in the study. This has been added to the methods: “Within the HDSS, participating household members’ demographics, educational level and households’ socio-economic quintile (relative to wealth across HDSS households) were previously recorded and last updated in 2019.”**

⎫ Please answer the following question very simply: Considering that the study includes people of all ages, are the educational level results for those over 18? (line152-153). **This is correct, we have now added this to the methods.**
⎫ Figure 1, can you explain why some people were not tested? **Added a note to the figure caption and to the methods (reasons for not being tested included participant absent, not possible to collect a swab, esp. in young children, or sample not correctly stored and transported).**
⎫ Please, include in all foot tables the name of the test used in the presented aOR, OR, HR (...) **Added to the title of Table 1 (it was already in Table 2 and 3).**
⎫ Table 1 - replace throat pain for sore throat. **Done**.
⎫ Figure 2, It is not very clear if “positive after vaccination” refers to vaccine-induced antibody +, please, consider replace the text for meaning is clear. **We added a note with definitions to the figure caption.**
⎫ Line 181 there is no need to parentheses. **Removed parentheses**
⎫
Discussion/Conclusion
⎫ Thanks for the discussion section, its drawn appropriately based on the data presented. **Thank you**
⎫ Please remove the parentheses of (symptomatic) on line 219, the sentence is confusing. **Removed**.
⎫ Review line 253. **Rephrased and adapted, linking to limitations.**
⎫ Did the authors observe any limitation? It would be nice to comment on in the discussion section. Please explain whether these limitations have been mitigated and how you plan to address them. Otherwise, authors can discuss any participation issue in the limitations section. **Added a subsection limitations**
⎫ There are few spelling errors that need to be revised. **Thank you, we have corrected all errors observed**

Reviewer #2: While interest in SARS-CoV-2 has waned with time, the manuscript provides valuable insight into trying to understand the impact of the pandemic in sub-Saharan Africa, and warrants publication. There are however, a few issues that need to be addressed:
1. It is unclear how participants were selected from the HDSS for the 2 weekly follow-ups, and what if any sampling strategy was used to recruit them. **Added a sampling strategy subsection in the methods**
2. An introduction to the HDSS population and description of the setting would be useful. My concern is that if the population were very homogeneous in terms of socioeconomic status, it would be difficult to pick up any differences in SARS-CoV-2 epidemiology with the current population size. **Indeed, we now added that to the limitations. We also provided a bit more detail on how households were selected from within the HDSS.**
3. The aHR values for socioeconomic quintiles and education in Table 3 are missing. These results are crucial to the findings of the study, and need to be reported. **Thank you for noticing, they are now added.**
4. Neighboring South Africa experienced a massive wave of SARS-Cov-2 infections in June/July 2020, and again in December 2020. As official case numbers under-counted the real extent of the pandemic, is it possible that Mozambique experienced mild SARS-CoV-2 in the middle of 2020, before the survey was started, and with the waning of antibody levels this "first" wave was missed? Either way, further interrogation of the lower seroprevalence results found in Maputo is needed, as the authors do not provide a sufficient explanation. The argument that prolonged NPI prevented transmission (line 233), might in true with regards to other neighbouring countries, but in the reference no 14 South Africa is noted to have stricter policies than Mozambique, contradicting the authors' explanation. **The limited spread before the Alpha wave in January 2021 as deducted from the low seroprevalence we observed in December 2020, was also supported by both ‘routine’ covid-19 surveillance, by bed capacity, and (anecdotal) absence from work by study staff. Even if only a limited proportion of cases were tested, nor PCR testing nor hospital bed capacity have been overwhelmed until January 2021, and only sporadically study staff has been absent due to symptoms prior to January 2021. Even if each evidence has its shortcoming, all three support the low sero-prevalence we observed at the start of the study. NPI might have been less stringent overall, international travel restrictions that were strictly followed and maintained over June-December 2020 might have limited seeding with imported COVID-19 cases. We rephrased and elaborated this part of the discussion to better reflect how those different elements support the low SARS-CoV-2 sero-prevalence.**
5. Line 252-253: "This discrepancy would be in line with observed lower pathogenicity in other sSA studies" - reference is needed. **We changed how we phrased this initially (quite hypothetically) as we think there is actually no consensus in evidence supporting that statement. E.g.** [**https://doi.org/10.1016/S0140-6736(24)00476-8**](https://doi.org/10.1016/S0140-6736(24)00476-8) **not reporting any better age-adjusted mortality in LMIC - even though inequity in distribution of vaccines is probably explaining some of the higher mortality rates observed in LMIC**
6. Line 247-248: No relationship between HIV and COVID-19 death was found. But how was HIV status ascertained? Was it only through respondent self-reporting, in which case exposure status misclassification might underestimate the relationship. I am not sure what the current HIV prevalence for adults in Mozambique is, but presume it is higher than the 7.5% reported amongst study participants. **Indeed, self-reported HIV status probably provides an underestimation of the actual prevalence (about double in Maputo City), and we have been careful how to phrase this. Nevertheless, even if misclassification could partially dilute the association, the effect size of our central estimate (below or around 1) does not indicate there is an association. We emphasized potential underestimation due to underreporting, explicitly for HIV, in the limitations (lines 306-308).**
